# Longitudinal changes in the frequency of mosaic chromosome Y loss in peripheral blood cells of aging men varies profoundly between individuals

**DOI:** 10.1038/s41431-019-0533-z

**Published:** 2019-10-25

**Authors:** Marcus Danielsson, Jonatan Halvardson, Hanna Davies, Behrooz Torabi Moghadam, Jonas Mattisson, Edyta Rychlicka-Buniowska, Janusz Jaszczyński, Julia Heintz, Lars Lannfelt, Vilmantas Giedraitis, Martin Ingelsson, Jan P. Dumanski, Lars A. Forsberg

**Affiliations:** 1 Department of Immunology, Genetics and Pathology and Science for Life Laboratory, Uppsala University, Uppsala, Sweden; 2 Faculty of Pharmacy and 3P Medicine Laboratory, International Research Agendas Programme, Medical University of Gdańsk, Gdańsk, Poland; 3 Department of Urology, Maria Sklodowska-Curie Memorial Cancer Centre and Institute of Oncology, Kraków Branch, Kraków, Poland; 4 Department of Public Health and Caring Sciences/Geriatrics, Uppsala University, 751 85 Uppsala, Sweden; 5 Beijer Laboratory of Genome Research, Uppsala University, Uppsala, Sweden

**Keywords:** Genetics, Genetic techniques

## Abstract

Mosaic loss of chromosome Y (LOY) is the most common somatic genetic aberration and is associated with increased risk for all-cause mortality, various forms of cancer and Alzheimer’s disease, as well as other common human diseases. By tracking LOY frequencies in subjects from which blood samples have been serially collected up to five times during up to 22 years, we observed a pronounced intra-individual variation of changes in the frequency of LOY within individual men over time. We observed that in some individuals the frequency of LOY in blood clearly progressed over time and that in other men, the frequency was constant or showed other types of longitudinal development. The predominant method used for estimating LOY is calculation of the median Log R Ratio of probes located in the male specific part of chromosome Y (mLRRY) from intensity data generated by SNP-arrays, which is difficult to interpret due to its logarithmic and inversed scale. We present here a formula to transform mLRRY-values to percentage of LOY that is a more comprehensible unit. The formula was derived using measurements of LOY from matched samples analysed using SNP-array, whole genome sequencing and a new *AMELX*/*AMELY*-based assay for droplet digital PCR. The methods described could be applied for analyses of the vast amount of SNP-array data already generated in the scientific community, allowing further discoveries of LOY associated diseases and outcomes.

## Introduction

Mosaic loss of chromosome Y (LOY) refers to chromosome Y aneuploidy acquired during lifetime and it is the most common post-zygotic variant described in human blood cells, causing the absence of almost 2% of the haploid nuclear genome [1]. For over 50 years it has been known that LOY is frequent in cells of the hematopoietic system [2], but LOY in leukocytes was long viewed as a neutral event related to normal aging without phenotypical consequences [3]. However, recent studies suggest that the opposite as LOY has been found to be associated with increased risk for all-cause mortality [4, 5] as well as a growing list of disease such as various forms of cancer [4, 6–10], autoimmune conditions [11, 12], Alzheimer’s disease [13], major cardiovascular events [5, 14], schizophrenia [15], diabetes [5] as well as age-related macular degeneration (AMD) [16]. As a male specific genetic risk factor for common disease, LOY in leukocytes might help explain why men live shorter lives [1, 4, 13, 17], likely as an effect of compromised immune system functions in circulating immune cells without chromosome Y [18].

In single cells LOY is a binary event, and when measured in bulk DNA samples collected from peripheral blood, it is manifested as a continuous mosaicism ranging from zero to 100% of cells without a Y chromosome. Recent studies have established that the frequency of LOY in leukocytes increases with age, occurring in at least 10% of peripheral blood cells in about 5–10%, 15–20% and 20–40% of aging men around 60, 70 and 80 years of age, respectively [4, 8, 13, 19–21]. Furthermore, in a cohort of 93 year old men, 57% of the individuals had lost the Y chromosome in more than 10% of the leukocytes [22]. Although age is a strong risk factor, occurrence and phenotypic effects associated with LOY in blood cells have also been reported in younger men [9, 16, 20]. LOY has also been described in other non-cancerous tissues, such as ectodermally-derived buccal mucosa [22] and atherosclerotic plaque [14], although typically in lower frequency than in haematopoietic cells. In addition to age, known risk factors for LOY during lifetime include smoking [8, 16, 19, 23], exposure to air pollution [24] as well as genetic background [8, 19, 21]. Further studies are needed for additional insights regarding its variation between individuals, its prevalence in different tissues, its changes in frequency over time within tissues (and in different cell populations within tissues) as well as its functional and phenotypic effects during the entire lifespan of men.

Measurements of LOY mosaicism from DNA can be performed using technologies such as karyotyping, qPCR, DNA-arrays and next generation sequencing (NGS) (Supplementary Table 1). Recent studies have successfully reanalysed data generated with SNP-arrays in various genome wide association studies (GWAS) and described profound phenotypic effects associated with LOY in leukocytes [4, 5, 8, 10, 13, 14, 16]. During the last decades, several large-scale genome projects have characterised the human genome and generated data that are suitable for analysis of occurrence of somatic structural variants and aneuploidies. Hence, available data could be reanalysed to further investigate associations between LOY (and other disease-associated somatic mutations/variants) in leukocytes with various human diseases and outcomes.

LOY mosaicism is straightforward to quantify from SNP-array data by calculation of a continuous variable called mLRRY, as further explained in the Methods and elsewhere [4]. The mLRRY-value in subjects without LOY are close to zero and it decreases with increasing level of LOY mosaicism and this relationship is a shortcoming for intuitive interpretation of the mosaicism. To solve this problem, we present here a formula to transform mLRRY-values into a more intuitive unit, i.e. the percentage of cells with the aneuploidy. We applied this transformation in comprehensive analyses of serially collected samples from aging men to characterise a previously unknown intra-individual variation of changes in the frequency of LOY within the blood of individuals studied over time. We further present a new effective method for estimation of LOY mosaicism based on quantification of the relative number of X and Y chromosomes using droplet digital PCR (ddPCR). The assay is targeting a 6 bp sequence difference present between the *AMELX* and *AMELY* genes using TaqMan-probes.

## Materials and methods

### Samples and DNA extraction

We analysed DNA from peripheral blood samples collected from participants of the cohort Uppsala Longitudinal Study of Adult Men (ULSAM, www.pubcare.uu.se/ulsam). For the longitudinal analyses, all available serially collected samples from ULSAM were included. This dataset comprised of 798 DNA samples collected from 276 men (median age = 81.9, range = 70–93) sampled 2–5 times over a period of up to 22.2 years (Supplementary Fig. 1). Furthermore, 121 DNA samples collected at 93 years of age from a subset of ULSAM participants were used for the pairwise analyses of LOY using three independent technologies (Supplementary Fig. 1). DNA was extracted from samples of peripheral blood nucleated cells using the QIAamp DNA Blood kit (51194, Qiagen) according to the manufacturer’s instructions. The study had been approved by the Regional Ethical Committee in Uppsala, Sweden (reference numbers, i.e. dnrs: 02-018, 02-605, 2007/338 and 2013/350). All study participants provided written informed consent.

### Measurements of LOY using three independent technologies

For comparing LOY measurements from different methods, we analysed the same set of DNA samples using three technologies, i.e. SNP-arrays (*n* = 121), whole genome sequencing (WGS, *n* = 26) and droplet digital PCR (ddPCR, *n* = 121). Description of how LOY estimations were performed using each readout is provided below. Briefly, for SNP-array data, the mLRRY variable was calculated as a median intensity of the probes located in the male-specific region of chromosome Y (MSY). For WGS, the frequency of cells with LOY was estimated from the ratio between the read depth on chromosome Y in relation to the full genome. For ddPCR, quantification of the relative number of X and Y chromosomes was performed by targeting a 6 bp sequence difference present between the *AMELX* and *AMELY* genes.

### LOY from SNP-array data

The estimation of level of LOY mosaicism from SNP-array data was performed by calculation of the mLRRY from data generated by Illumina BeadChips (Illumina Inc., CA, USA) as previously described [4]. For each experiment passing strict quality control [13], the mLRRY-value was calculated as the median of the Log R Ratio (LRR) of the probes located in the MSY (chrY: 2.781.480–56.887.902, hg19/GRCh38.p12). The different versions of arrays used each contain sufficient number of probes for robust calculation of mLRRY (Supplementary Table 2). The mLRRY-values calculated for every sample was corrected for batch effects by adjustment using the local regression median of mLRRY-values from a kernel density estimation, using the density function in R on a smooth histogram of mLRRY-values, as previously described [4].

### LOY from whole genome sequencing (WGS) data

Estimation of LOY mosaicism from WGS data was performed by comparing the read depth on chromosome Y in relation to the full genome. Sequencing libraries were prepared using the truseq Nano DNA sample preparation kit (T FC-121- 4001/4002, Illumina Inc) extracting 100 ng DNA for each sample. Sequencing libraries were run on an Illumina HiSeq X instrument (version 2.5 sequencing chemistry) and sequenced to a depth of 30×. Each sequenced library had a read length of 150 bp with an insert size of 350 bp. Sequencing reads were aligned to the GRCh37 human reference genome with the BWA aligner (version 0.7.12). Copy number for chromosome Y was estimated by the Control-Freec software using read counts in non-overlapping windows across the genome. These were fitted by the GC content and mappability information and the median ploidy for the Y chromosome was calculated [25].

### LOY from droplet digital PCR (ddPCR) data

Estimation of LOY with ddPCR was performed using Bio-Rad’s QX200 Droplet Digital PCR System (Bio-Rad Laboratories, Inc., CA, USA). A TaqMan-based method was developed and used to quantify the relative number of X and Y chromosomes in a sample by targeting a 6 bp sequence difference present between the *AMELX* and *AMELY* genes (Supplementary Fig. 2). An advantage of this protocol compared with previous qPCR-based methods, is that the PCR amplification of the two target-genes (i.e. the test and reference loci on chromosomes Y and X, respectively) are performed using the same primer pair and would thus be relatively unbiased with regard to primer-properties. Primers and probes was purchased from Thermo Fisher Scientific (MA, USA) article number C_990000001_10. DNA samples with concentrations ranging between 300 and 20 ng/µl were digested for 15 min in 37 °C with HindIII (Thermo Fischer, article number: #FD0504) and diluted with an equal volume of water. Subsequently 50 ng of the digested and diluted DNA sample was mixed in PCR supermix for probes without dUTP (BioRad, article number: 186–3023) together with TaqMan primers and probes. Following manufacturer’s instructions, droplets were generated and PCR amplified using the following conditions: 95 °C for 10 min, 40 cycles of 94 °C for 30 s and 60 °C for 1 min. The PCR programme ended with 98 °C for 10 min and a 10 °C hold. The fluorescence of FAM (targeting *AMELY*) and VIC (for *AMELX*) was analysed for each droplet using a droplet reader and the ratio *AMELY*/*AMELX* was analysed using Bio-Rad’s software QuantaSoft (version 1.7.4.0917). All samples were run in duplicates and the standard deviation of the measured ratios was calculated. Samples were re-analysed if the standard deviation was 1.2 or higher.

### Transformation of mLRRY-values into percentage of cells with LOY

We developed a formula for conversion of mLRRY-values estimated from SNP-array data into the unit LOY (%) by taking advantage of LOY-estimates from pairwise studied DNA samples. First we determined that the percentage of LOY estimated in samples studied by WGS and ddPCR (*n* = 26) yielded essentially identical results. The readouts from these technologies could therefore be used as reference points for establishing a relationship between mLRRY and LOY (%). The latter was performed in parallel and independently using the data generated from pairwise studied samples using ddPCR and SNP-array (*n* = 121) as well as from samples analysed using WGS and SNP-array (*n* = 26) (Supplementary Fig. 3). Specifically, the mLRRY-values were first antiloged (2^mLRRY^) and correlated with data generated from the same samples by WGS (*n* = 26) or ddPCR (*n* = 121). Calculation of power equations resulted in the relationships *y* = 0.9242*(2^*x*)^1.7703 and *y* = 0.9695*(2^*x*)^1.8779 (describing the relationships between level of LOY estimated by mLRRY and percentage of LOY estimated by WGS or ddPCR, respectively). The constants in these relationships were rounded to the nearest integer and used to adjust the antilog mLRRY, resulting in two equations: (1) *percent of cells with a Y chromosome* = 100*2^2*mLRRY^ and (2) *LOY (%)* = 100*(1−2^2*mLRRY^).

### Statistical analyses

Calculations of Pearson’s coefficient of determination (*R*^2^) and linear regression models were performed using *R*. Standardised beta-values (*β*) was calculated using the R library lm.beta [26].

## Results

### Longitudinal analyses of LOY frequency in peripheral blood of 276 aging men

**Fig. 1:**
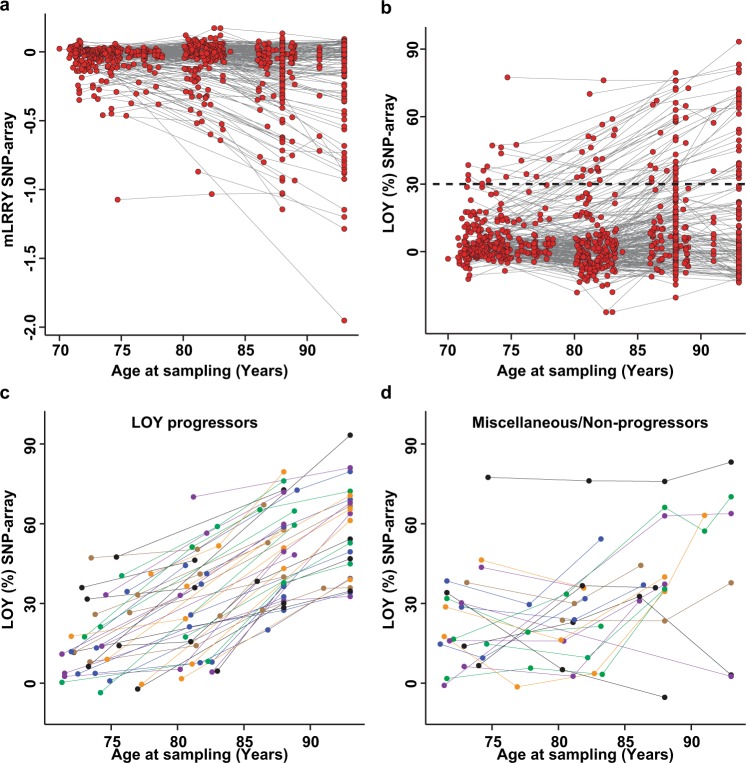
Results from longitudinal analyses of LOY mosaicism in whole blood DNA from 276 aging individuals from the ULSAM cohort. Subjects were sampled serially 2–5 times over a period of up to 22.2 years. Every point represents a measurement of LOY in a subject at one time point with the level of LOY estimated from SNP-array data at the Y-axes and age of sampling on the X-axes. The dataset before (**a**) and after transformation (**b**) of the mLRRY-values using the equation: LOY (%) = 100 × (1−2^2*m*LRRY^). Grey lines connect the LOY measurements from the same individual at different time points. The dotted black line in **b** indicates a level of LOY mosaicism at which at least 30% of the nucleated blood cells are without a Y chromosome. This threshold was used to identify subjects displaying a high level of LOY at any time point during the study and the longitudinal changes in the frequency of LOY in this subset are displayed in **c** and **d**. To visualise changes in LOY frequency over time, points and lines were colour-coded to connect multiple measurements from the same individual. **c** Displays the subjects showing a clear progression in the frequency of LOY over time and **d** shows non-progressing individuals with miscellaneous types of longitudinal change

Analyses of the serially collected samples in the ULSAM study showed an overall higher level of LOY mosaicism in samples collected at higher ages (Fig. 1). The age-related accumulation of LOY was significant in linear regression models using the continuous mLRRY as response variable (*β* = −0.21, *p* < 0.0001) as well as using the new LOY (%) unit (*β* = 0.19, *p* < 0.0001). Furthermore, the serial analysis revealed a previously undescribed profound inter-individual variation in the changes of frequency of LOY in blood over time. For example, in about 1/3 of the individuals, the level of LOY mosaicism clearly increased with age, i.e. LOY progressors. In other subjects, the level did not change substantially during the follow-up time (Fig. 1b–d). Furthermore, in a few subjects the level of mosaicism decreased during the study and more complex and miscellaneous patterns were also observed, such as initial increase followed by a decrease but also the reverse with an initial decrease followed by increased level of mosaicism (Fig. 1d). The dotted line in panel Fig. 1b marks a threshold where 30% of the blood cells are without the Y chromosome and we identified 65 individuals that in at least one time point had a level of LOY on or above this threshold. In this subset, an increased level of LOY over time could be observed in a majority of subjects (Fig. 1c) while other subjects displayed different patterns (Fig. 1d). The longitudinal changes in the frequency of LOY mosaicism within each of the 276 studied individual subjects is provided in Supplementary Fig. 4.

### New formula for transformation of mLRRY to percentage of LOY

**Fig. 2:**
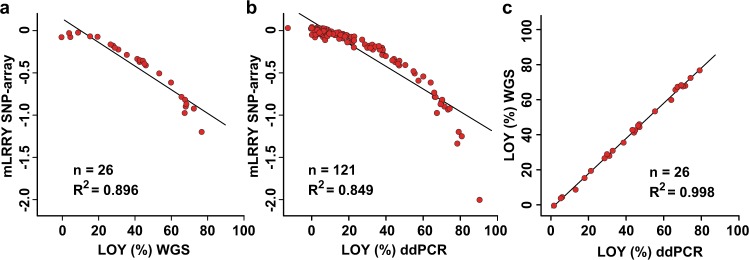
Illustration of the non-linear estimation of LOY mosaicism by mLRRY calculated from SNP-array data by comparisons with LOY estimates generated from the same set of samples using independent technologies. **a**, **b** show the comparisons between mLRRY calculated from SNP-array data with the corresponding LOY estimates generated from the pairwise studied samples using whole genome sequencing (WGS) and droplet digital PCR (ddPCR), respectively. **c** displays a high concordance between estimates of LOY in samples pairwise studied with WGS and ddPCR. A linear regression line with Pearson’s coefficient of determination (*R*^2^) is shown for each comparison

**Fig. 3:**
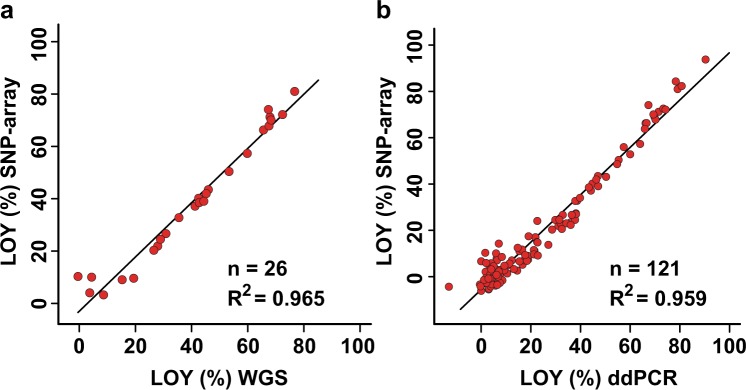
Transformation of mLRRY-values linearises LOY estimates from SNP-array data using the equation: LOY (%) = 100 × (1−2^2*m*LRRY^). **a** displays estimations of the level of LOY from 26 pairwise studied samples using SNP-array and whole genome sequencing (WGS). The *Y*-axis show the predicted LOY (%) from SNP-array data using the above formula and the X-axis display the measured level of LOY using WGS. A linear regression line with Pearson’s coefficient of determination (*R*^2^) is shown. **b** shows a corresponding comparison between estimations of LOY from 121 pairwise studied samples using SNP-array and droplet digital PCR (ddPCR)

We used data generated by SNP-array, whole genome sequencing (WGS) and droplet digital PCR (ddPCR targeting a sequence difference between the *AMELY* and *AMELX* genes) to estimate the level of LOY in DNA from whole blood samples. The LOY estimate from each experiment is provided in Supplementary Table 3. This dataset of pairwise analysed samples made it possible to compare measurements of LOY in individual samples estimated using the three independent technologies (Fig. 2). These comparisons exposed a non-linear estimation of LOY using the mLRRY calculated from SNP-array data (Fig. 2a, b) as well as a linear estimation of LOY by WGS and ddPCR. Specifically, a close to perfect fit to a linear regression line was observed between readouts from the same samples using WGS and ddPCR (Pearson’s coefficient of determination, *R*^2 ^= 0.998, *n* = 26) (Fig. 2c). Comparing the level of LOY estimated in the samples analysed using SNP-array and WGS (*n* = 26) as well as in the samples analysed using SNP-array and ddPCR (*n* = 121) also showed concordance with respect to level and direction of mosaicism (Fig. 2a, b). However, in contrast to the linear correlation between WGS and ddPCR readouts, a non-linear relationship (likely due to the logarithmic scale of the LRR-values) was observed and the fit to a linear regression line between the readouts from these technologies and mLRRY from SNP-arrays was lower (*R*^2 ^= 0.896 and 0.849, respectively). Transformation of mLRRY-values into percentage of LOY using the equation LOY (%) = 100*(1−2^2*m*LRRY^) resulted in improved fit to linearity (*R*^2 ^=  0.965 and 0.959, respectively) (Fig. 3a, b).

**Fig. 4:**
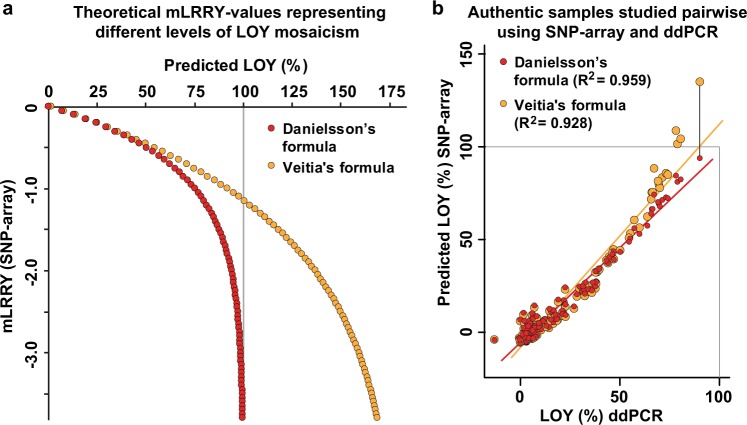
Comparison of the performance of two formulas developed for prediction of percentage of LOY from mLRRY-values, i.e. Danielsson’s formula presented here and the recently published Veitia’s formula. In **a**, the estimates of mosaicism from each formula are compared in a range of theoretically possible mLRRY-values representing varying degree of LOY. The values of mLRRY plotted on the *Y*-axis were used to predict the percentages of LOY plotted on the *X*-axis. In male subjects without LOY, mLRRY-values are close to zero and lower values indicate increasing level of LOY mosaicism. The mLRRY calculated from female samples typically range −3 to −4. The horizontal grey line at 100% represents an extreme level of mosaicism (Y loss in all cells) and thus indicates a maximum theoretical limit of predicted LOY mosaicism in men. **b** shows a similar comparison using authentic data generated from 121 samples studied with both SNP-array and ddPCR. The *Y*-axis shows the predicted percentage of LOY from SNP-array data using each formula and the level of mosaicism measured by ddPCR in corresponding samples are plotted on the *X*-axis. Grey lines indicate theoretical upper limits of LOY estimations. To illustrate the overestimation of LOY mosaicism generated by the Veitia’s formula, a black line connects the predictions from each formula in the sample with the highest level of LOY mosaicism

To evaluate the ability of the formula to predict biologically relevant levels of LOY mosaicism we first tested it using a range of theoretically possible mLRRY-values representing varying degree of LOY (Fig. 4a). For comparison, we also applied another published formula [27] on the same dataset (i.e. Veitia’s formula *F*_(LOY)_ = 1.8 (1−2^mLRR^) + 0.015). The evaluation showed that the two formulas generates similar predictions of mosaicism at low levels of LOY but at higher levels of mosaicism, only the formula presented here asymptotically approaches the theoretical maximum of 100% mosaicism. In Fig. 4b, the ability of the formulas are assessed using authentic datasets generated from 121 samples studied with both SNP-array and ddPCR. Also in these comparisons, Veitia’s formula tend to overestimate the level of LOY in samples with high levels of mosaicism, while the formula presented here predicts LOY (%) within relevant boundaries.

## Discussion

During human lifespan, somatic cells acquire various forms of post-zygotic genetic variants that occasionally mediates a proliferative advantage to affected cell(s) [1, 20, 28–37]. Such processes of cell expansion are often referred to as clonal haematopoiesis (CH or CHIP) when affecting blood cell progenitors. A more general term to describe such cellular progressions, useful for all types of cells and not restricted to events in the haematopoietic system, is aberrant clonal expansions (ACE) [1]. LOY in leukocytes is the most common form of ACE showing a clear increase in frequency with age in the general population [4, 8, 13, 19–22]. The serial sampling applied in the present study revealed a previously unknown and profound variation in the dynamics of LOY-clone evolution within the peripheral blood of individual subjects over time. The participants of ULSAM study have been followed clinically for almost 50 years and blood samples have been collected up to five times from the same participants during the last decades. We observed that in certain subjects, ACEs with LOY clearly progressed with time and that in others, the frequency of LOY cells did not change substantially with age or showed other types of LOY-clone trajectories (Fig. 1 and Supplementary Fig. 4). The possible mechanism(s) behind these profound differences between individuals is currently not known but could be related to variation in proliferative rates and longevity of progenitor cells giving rise to LOY-clones. Furthermore, variation in exposures to external risk factors (smoking, air pollution etc.) as well as other possible confounders (disease, therapies etc.) could explain part of the observed variation in progression patterns, a topic that require further studies. Moreover, some individuals in the longitudinal dataset showed indications of low-frequency mosaic gain of chromosome Y (GOY) at several measured time points (Fig. 1). It is not clear if all of these observations are of biological origin or represent technical variation. However, in one individual, GOY was detected using both SNP-array and ddPCR (Figs. 2b and 3b, i.e. subject 207 in Supplementary Fig. 4). To our knowledge, no phenotypic consequences from mosaic gain of chromosome Y in leukocytes has been described.

It should also be noted that the samples studied here was bulk DNA extracted from whole blood samples. It is therefore unknown which blood cell type(s) that were affected with LOY in the subjects of the dataset, and thus, if the observed variation in clonal trajectories are an effect from LOY in different cell types. However, results from a recent analysis [18] shows that the level of LOY varies substantially between different types of leukocytes in blood, with generally higher levels in myeloid compared with lymphoid lineages, and that LOY often occurs as an oligo-clonal event in peripheral blood. To further elucidate if and how the developmental trajectories of LOY-clones varies between the different types of leukocytes in blood, studies of the frequency of LOY in different cells types in serially collected subjects will be informative. Interestingly, we observed in a subset of the studied men that the frequency of LOY in whole blood samples increased in a non-linear fashion (Supplementary Fig. 4). Such expansions would likely be an effect of oligo or polyclonal processes, i.e. several hematopoietic progenitor cells giving rise to ACEs without the Y chromosome.

The predominant proxy used for estimation of LOY mosaicism is the mLRRY calculated from the median LRR-values of SNP-array probes positioned in the MSY [4]. Other methods to estimate LOY have also been proposed, such as calculation of the mean LRR of MSY-probes [8]. However, this approach could potentially be more sensitive to biases from probes in ampliconic regions compared with estimations using the median. Hence, the median would represent the average probe-intensity more accurately since the intensity values of outliers will have less weight on the calculated mLRRY. Furthermore, a data type called B allele frequency (BAF) is generated by SNP-arrays (in addition to SNP-calls and the LRR-data) and several algorithms have been developed for detection of autosomal mosaic chromosomal alterations using imbalances in BAF [28, 35, 37]. Recently, the BAF has also been used to estimate LOY mosaicism by mapping of BAF deviations in probes located in the pseudo-autosomal regions, a region that is shared between the X and Y chromosomes [21]. Although haplotype information is incorporated in this approach, it is currently not known if uncontrolled variation (such as potential X-mosaicism) could potentially influence the estimates of low level LOY mosaicism using this algorithm. In contrast, the mLRRY is calculated from Y-specific variation in the MSY. However, the mLRRY-estimate has other shortcomings, such as its logarithmic and inversed scale (Fig. 2). Here we present a formula to transform mLRRY-values from SNP-arrays to the unit percentage of cells with LOY, which is a more intuitive unit on a linearised scale. The formula for mLRRY-transformation was established by analysing the same DNA samples using three independent technologies, i.e. SNP-array, WGS and ddPCR. A detailed description of how LOY was estimated using each technology is provided in the Materials and Methods. We first established that LOY estimation using WGS and ddPCR yielded close to identical results from the same set of DNA samples (Fig. 2c). The LOY estimates from these technologies could therefore be used as a baseline to derive a formula for transformation of mLRRY to percentage of LOY. Of note, the same formula was independently derived from analyses of the WGS and ddPCR datasets, i.e. LOY (%) = 100*(1−2^2*mLRRY^). After transformation of mLRRY-values with this formula, the concordance of the SNP-array data improved substantially in comparisons with readouts from the other technologies (Fig. 3).

It should be noted that the results presented here are based on data generated by different versions of Illumina genotyping arrays (Supplementary Table 2). Further studies are needed to evaluate the performance of the formula using data generated from different arrays and from other manufacturers, for example by the here presented ddPCR approach. The unit percentage of LOY represents the studied biological event in a more comprehensible way compared with mLRRY, since a higher percentage of LOY is indicative of a higher level of mosaicism. We also established that the formula predicts the level of LOY mosaicism within a theoretically and biologically relevant continuum (between zero and 100% mosaicism) in contrast to a recently published formula [27] (Fig. 4).

In conclusion, we describe a dynamic nature of changes in the frequency of LOY within in the hematopoietic system of serially studied men. A group of individuals were identified as LOY progressors with a clear expansion over time, while in others, the longitudinal frequency remained unchanged or showed other types of trajectories. We also present a formula for transformation of mLRRY-values calculated from SNP-array data into percentage of LOY and describe a new TaqMan/ddPCR-based method for efficient LOY analysis. The pipelines and methods described will be useful to further investigate associations between LOY in leukocytes and various outcomes.

## Supplementary information

Supplementary Information
