# Supplementary material for: Longitudinal changes in the frequency of mosaic chromosome Y loss in peripheral blood cells of aging men varies profoundly between individuals
Source: Eur J Hum Genet. 2019 Oct 25;28(3):349–57. doi: 10.1038/s41431-019-0533-z (PMC7028735; doi:10.1038/s41431-019-0533-z)
Supplement: Supplementary file 1 — Supplementary Information [file 41431_2019_533_MOESM1_ESM.pdf]

Supplementary Information

Longitudinal changes in the frequency of mosaic chromosome Y loss in peripheral blood cells of aging men varies profoundly between individuals

Danielsson, Halvardson, Davies, Torabi Moghadam, Mattisson, Rychlicka-Buniowska, Jaszczyński, Heintz, Lannfelt, Giedraitis, Ingelsson, Dumanski and Forsberg

Item ***Page(s)***

Supplementary Figure 1………………...2

Supplementary Figure 2………………...3

Supplementary Figure 3………………...4-5

Supplementary Figure 4………………...6-15

Supplementary Table 1………………....16

Supplementary Table 2………………....17

Supplementary Table 3………………....18-20

Supplementary Figure 1.

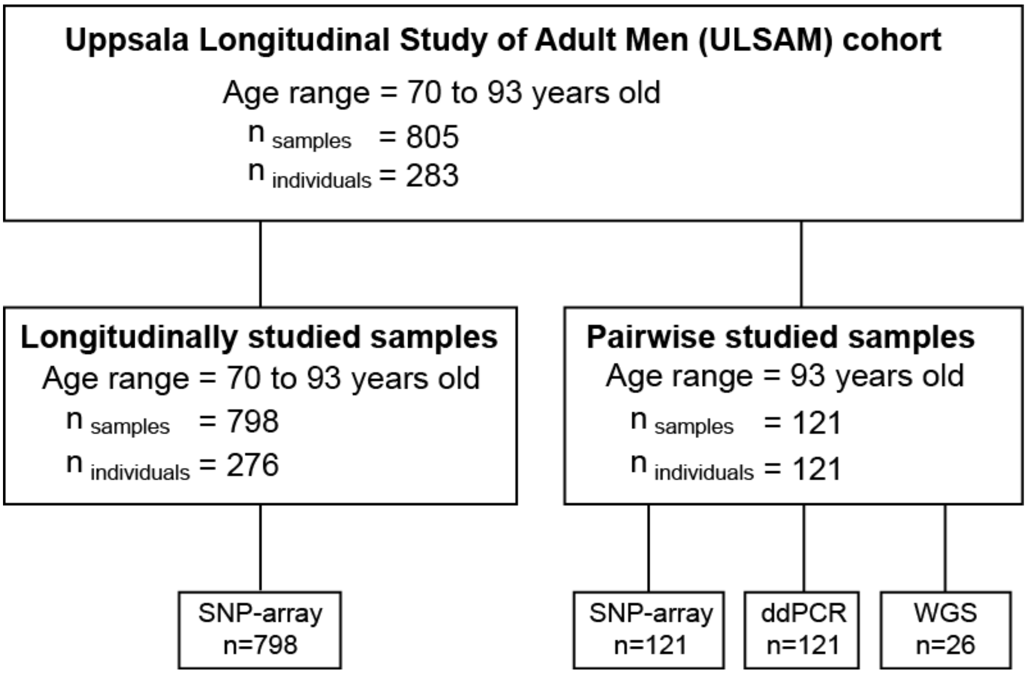

Supplementary Fig. 1. Description of the samples from the Uppsala Longitudinal Study of Adult Men (ULSAM) included in the present study. For the analyses of longitudinal changes, we used serially collected samples. Furthermore, LOY estimates from different technologies was compared in a subset of the cohort from samples analyzed with SNP-array, droplet digital PCR (ddPCR) and whole genome sequencing (WGS). The number of individuals, number of samples analyzed with each technology as well as age range of individuals at sampling is specified.

Supplementary Figure 2.

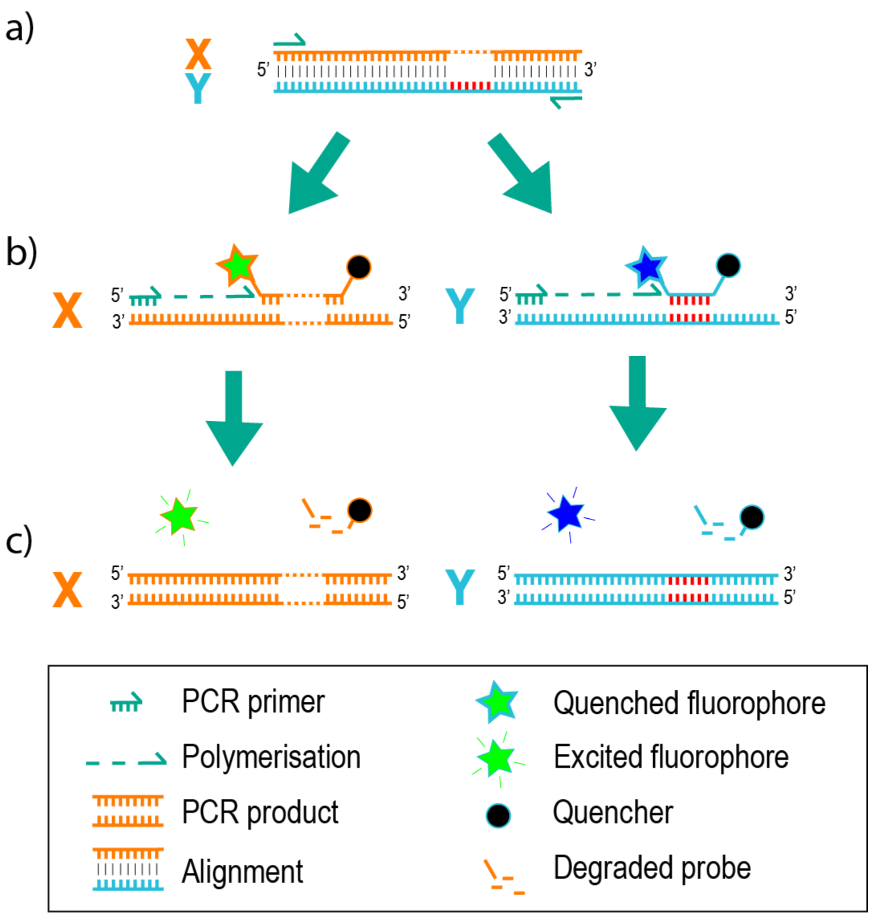

Supplementary Fig. 2. Principal view of the *AMELY*/*AMELX* TaqMan assay for efficient LOY estimation from DNA samples. The target for this assay is a 6-bp polymorphism between the *AMELX* and *AMELY* genes on chromosome X and Y, respectively (**a**). The loci containing the 6-bp deletion is targeted for PCR-amplification using identical forward and reverse primers for *AMELX* and *AMELY* (**a**). During the PCR-amplification, fluorescently labelled (and quenched) TaqMan probes specifically hybridise to either the deleted site on *AMELX* or the inserted site on AMELY (**b**). The fluorophores are cleaved of by the polymerase, allowing excitation of the fluorophore (FAM for *AMELY* and VIC for *AMELX*) (**c**).

Supplementary Figure 3.

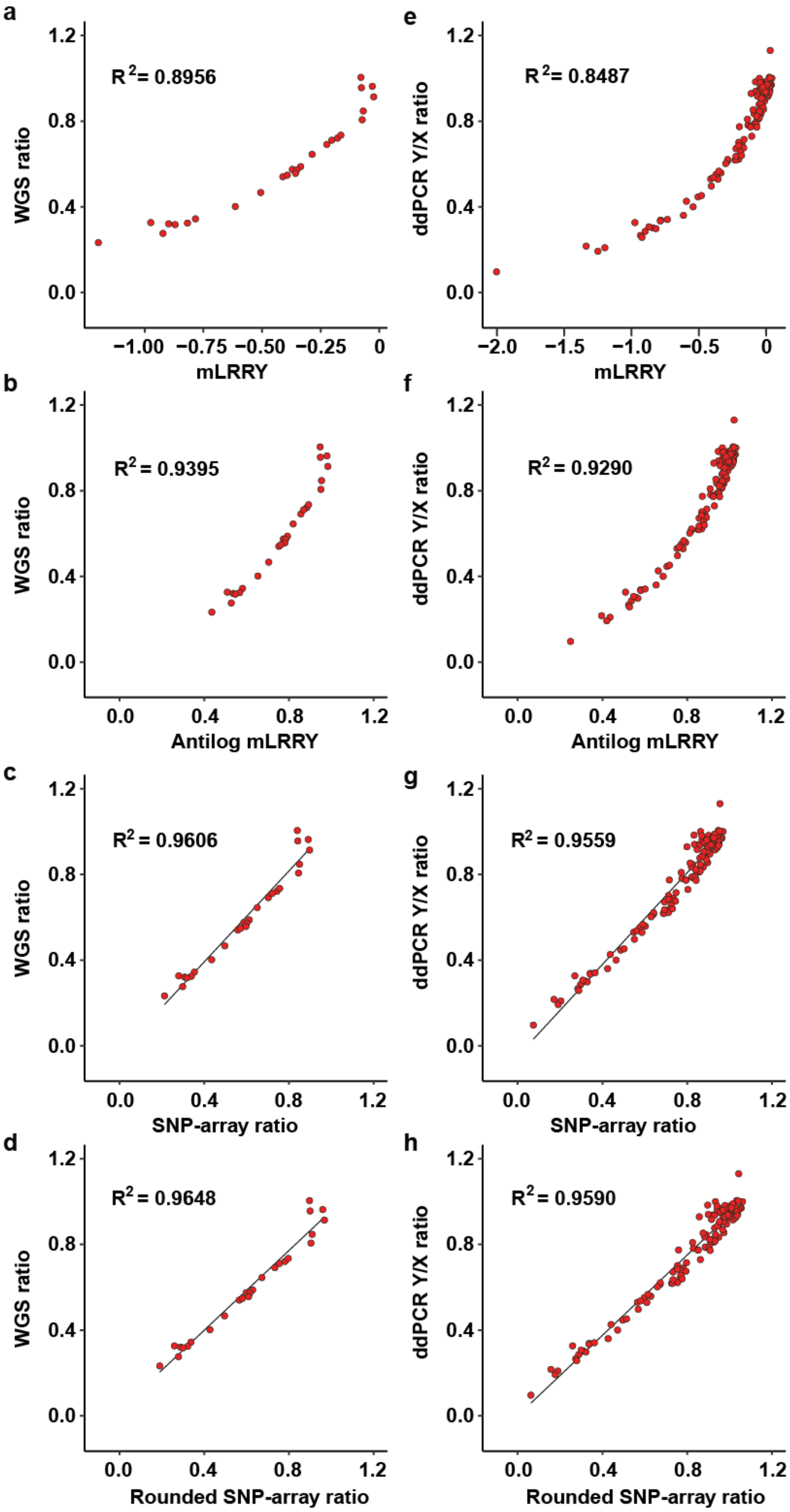

Supplementary Fig. 3. Transformation of mLRRY-values to percentage LOY using empirical data generated by independent technologies. Panels **a-d** represents the steps performed to transform mLRRY to LOY (%) using the baseline measurements generated in the pairwise studied samples analysed with whole genome sequencing (WGS). The corresponding derivations using ddPCR data is shown in panels **e-h**. First, panel **a** show the ratio of XY-cells in 26 samples analysed by WGS and the corresponding mLRRY-values estimated using SNP-array data from the same DNA samples. In panel **b**, the antilog of mLRRY (2^{mLRRY}) was plotted on the x-axis and the power equation $Y=0.9242 \times (2^{X})^{1.7703}$) was calculated. By transforming the mLRRY according to this equation a ratio of XY-cells could be generated and plotted on the x-axis in panel **c**. In the final formula the constants were rounded to the closest integer ($Y=2^{2 \times X}$) and plotted on the x-axis in panel **d**. An analogous transformation of the mLRRY using data generated with ddPCR (n=121) resulted in the power equation $Y=0.9186 \times (2^{X})^{1.8138}$ (panels **e-h**) and hence, after rounding to closest integer, the same formula was derived by comparison with two independent technologies, i.e. *LOY (%)* = $100*(1-2^{2*mLRRY})$.

Supplementary Figure 4. (part 1/10)

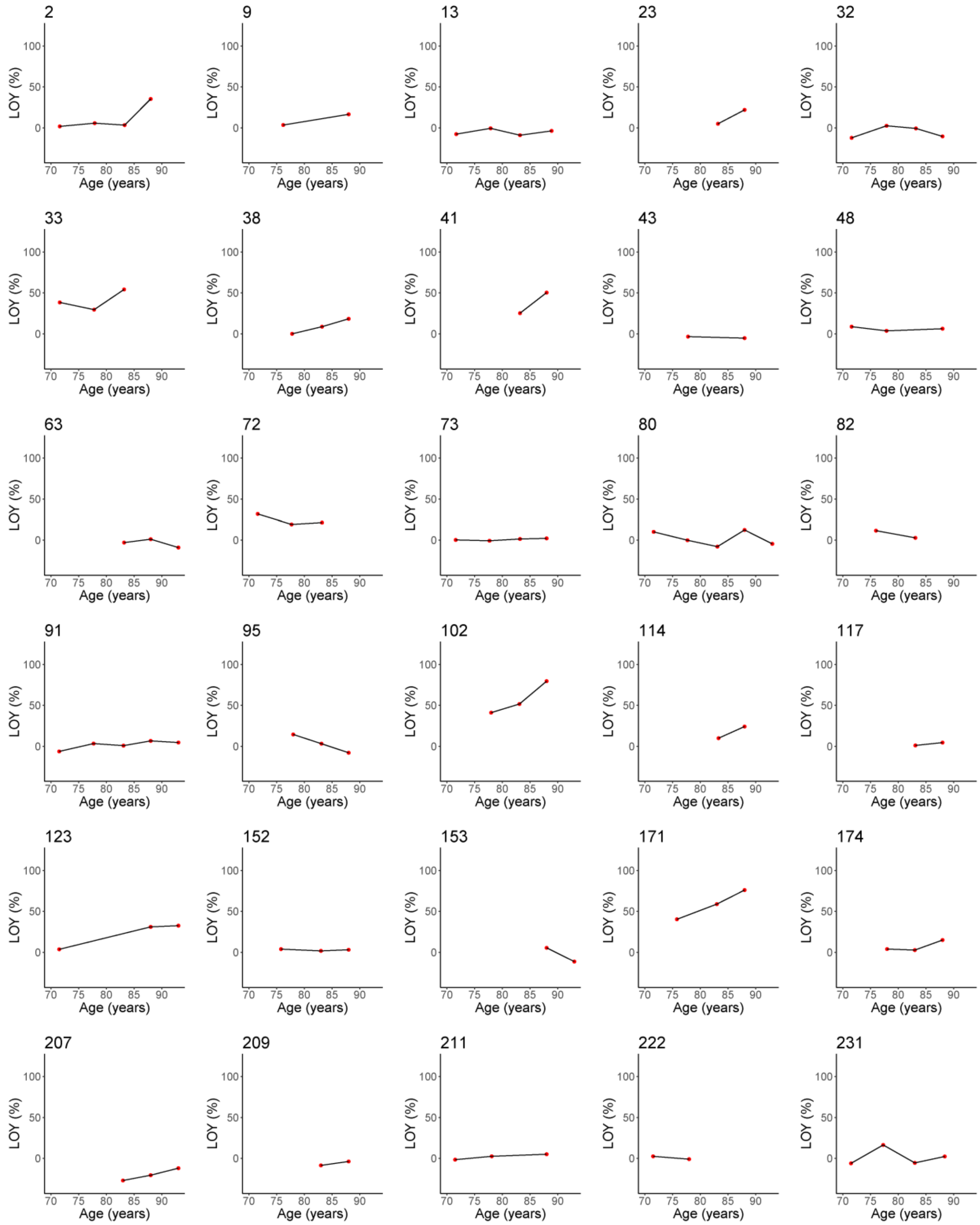

Supplementary Figure 4. (part 2/10)

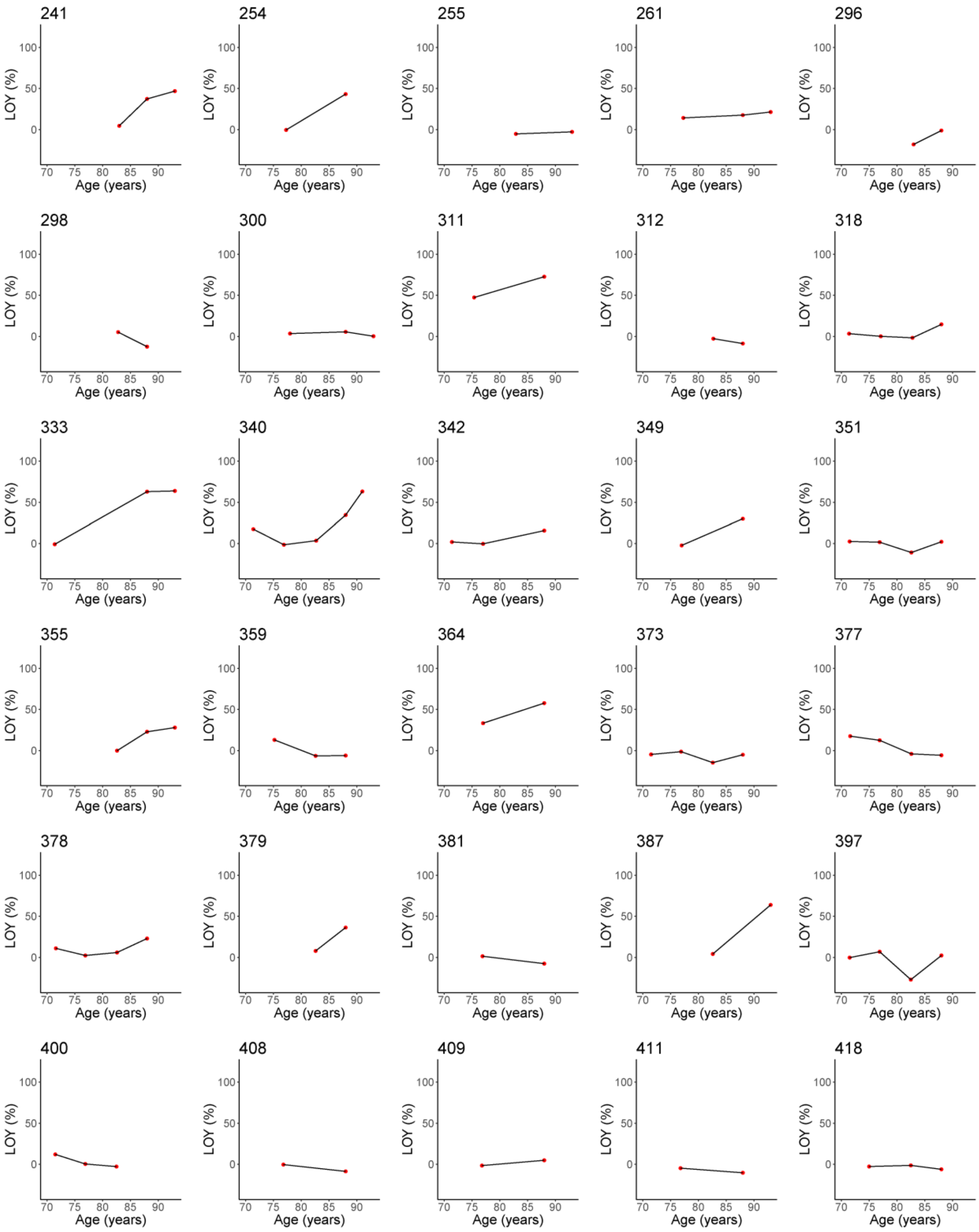

Supplementary Figure 4. (part 3/10)

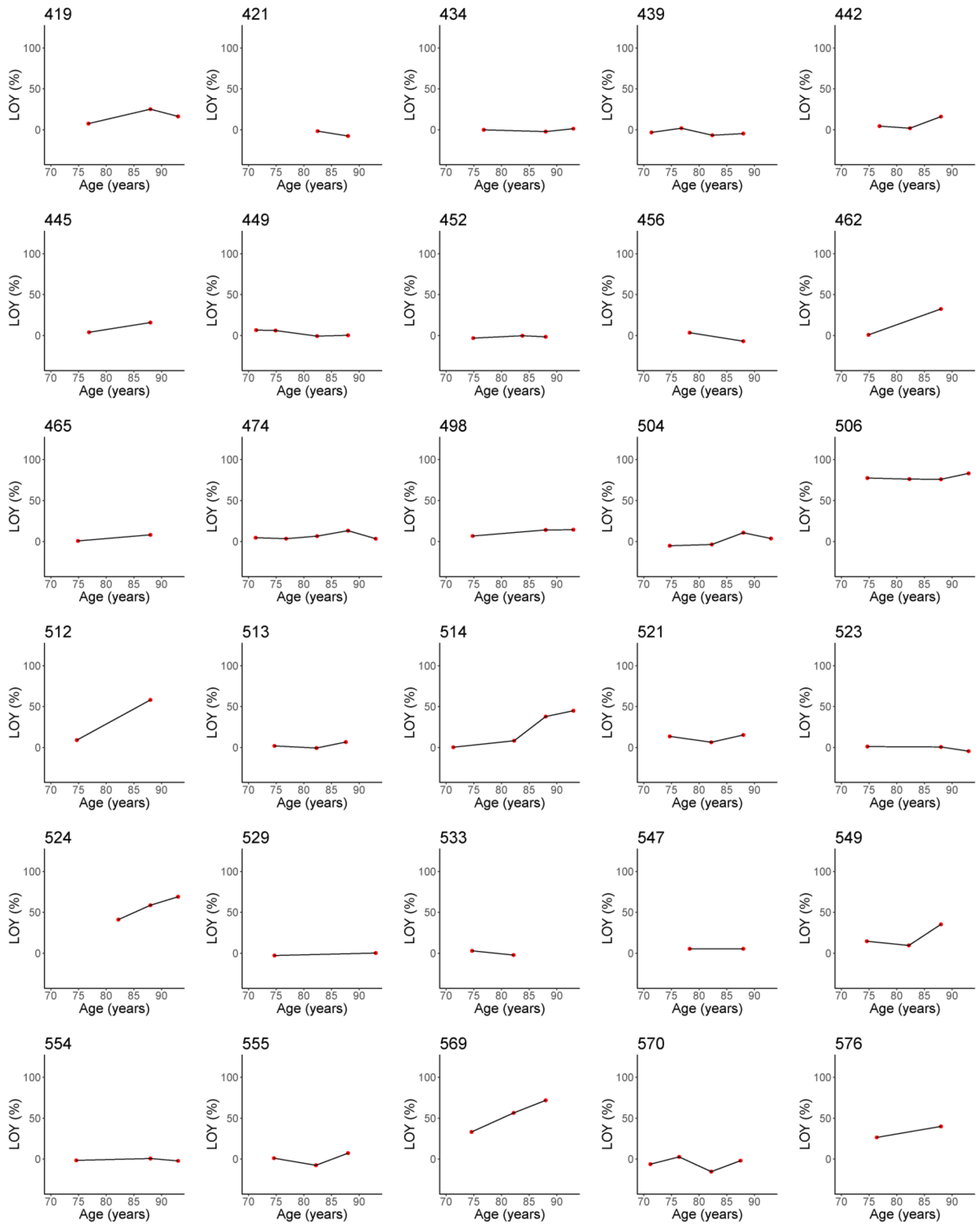

Supplementary Figure 4. (part 4/10)

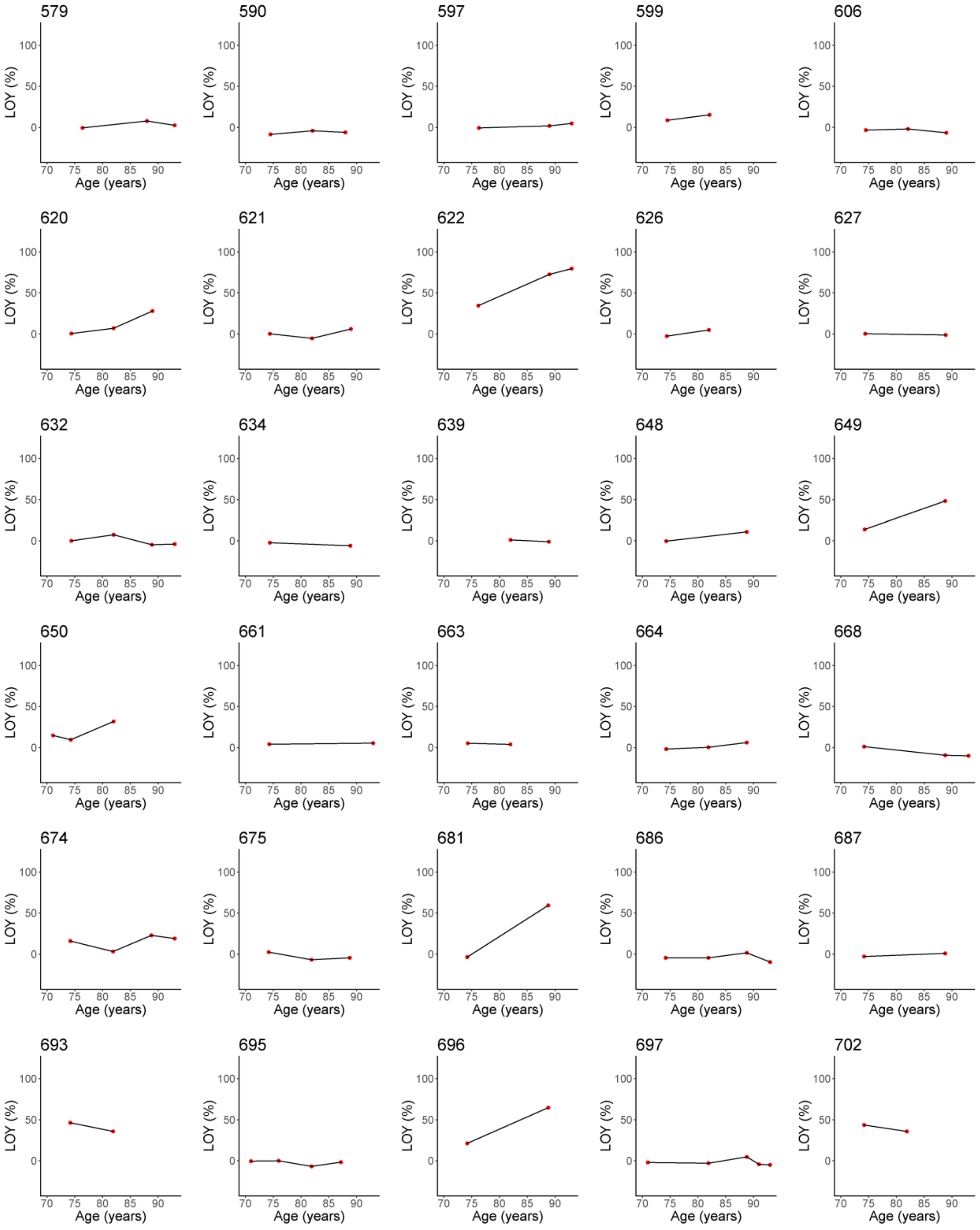

Supplementary Figure 4. (part 5/10)

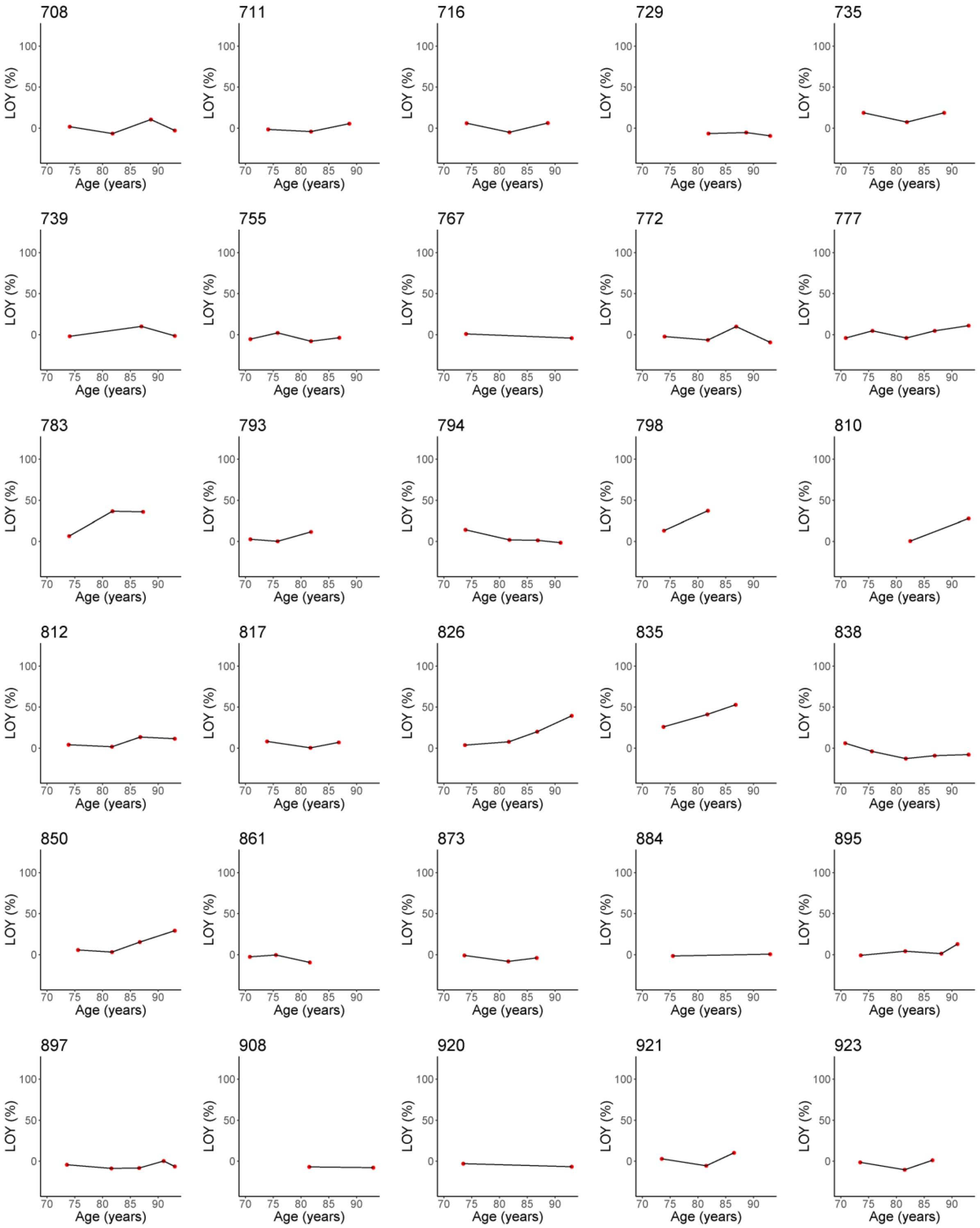

Supplementary Figure 4. (part 6/10)

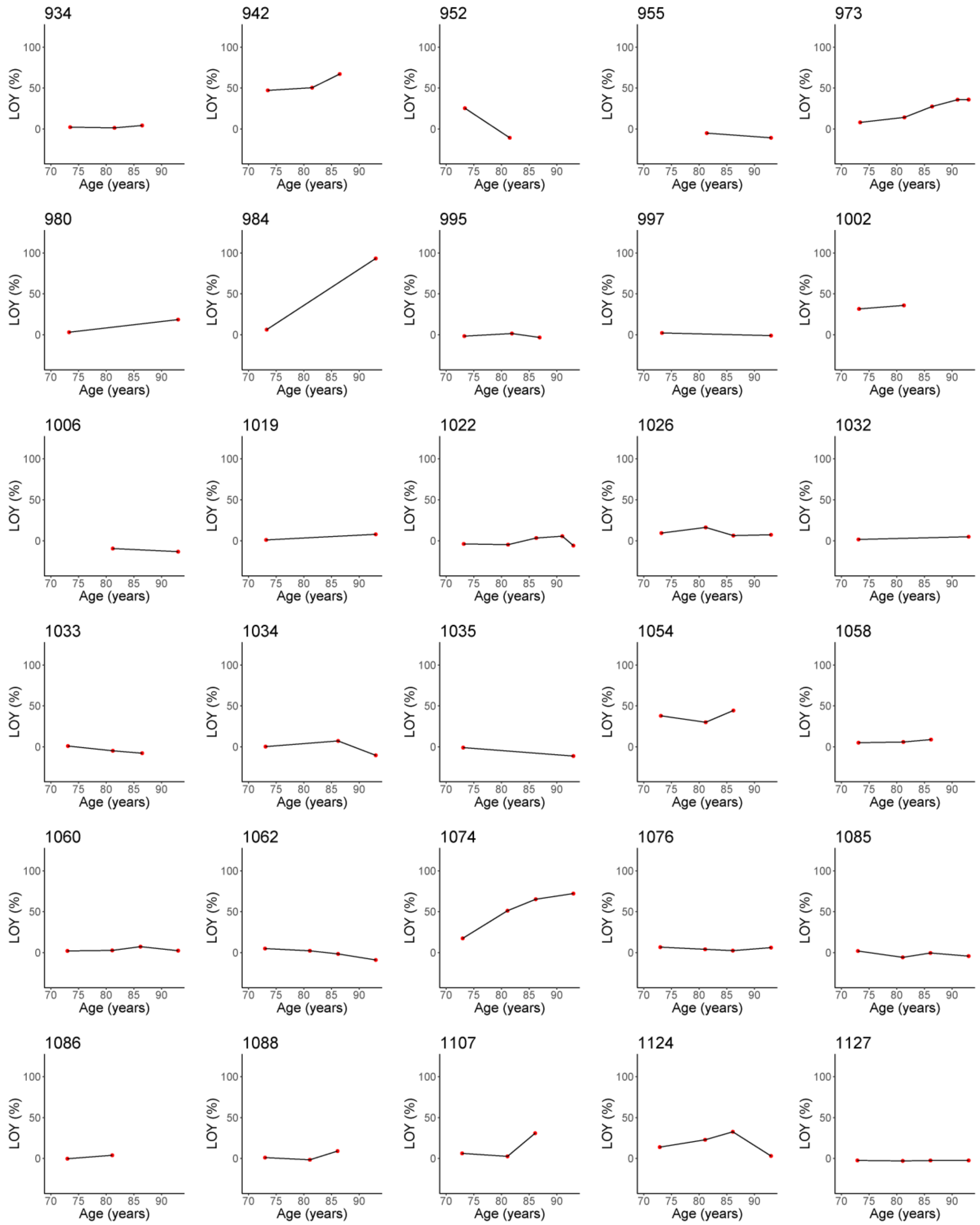

Supplementary Figure 4. (part 7/10)

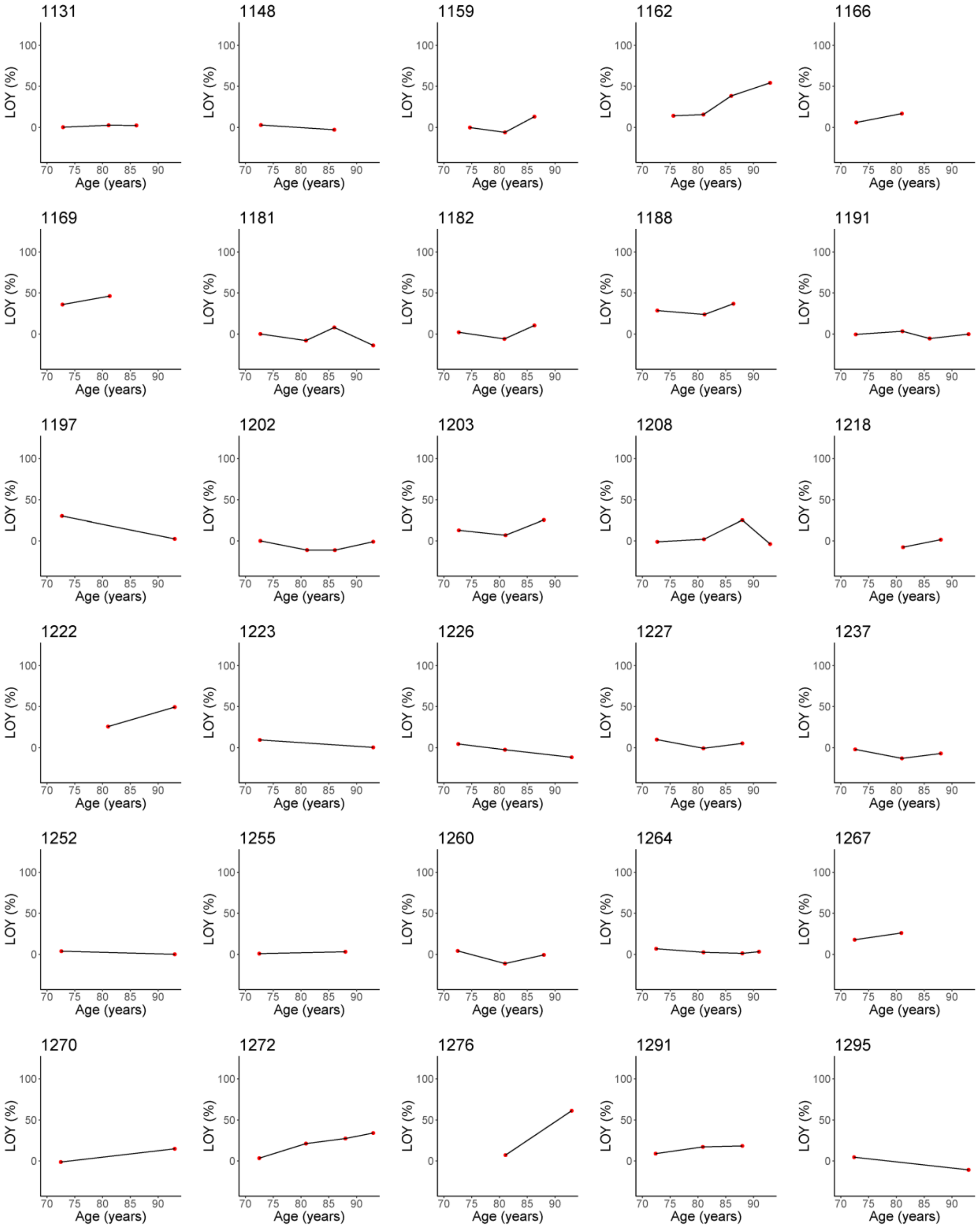

Supplementary Figure 4. (part 8/10)

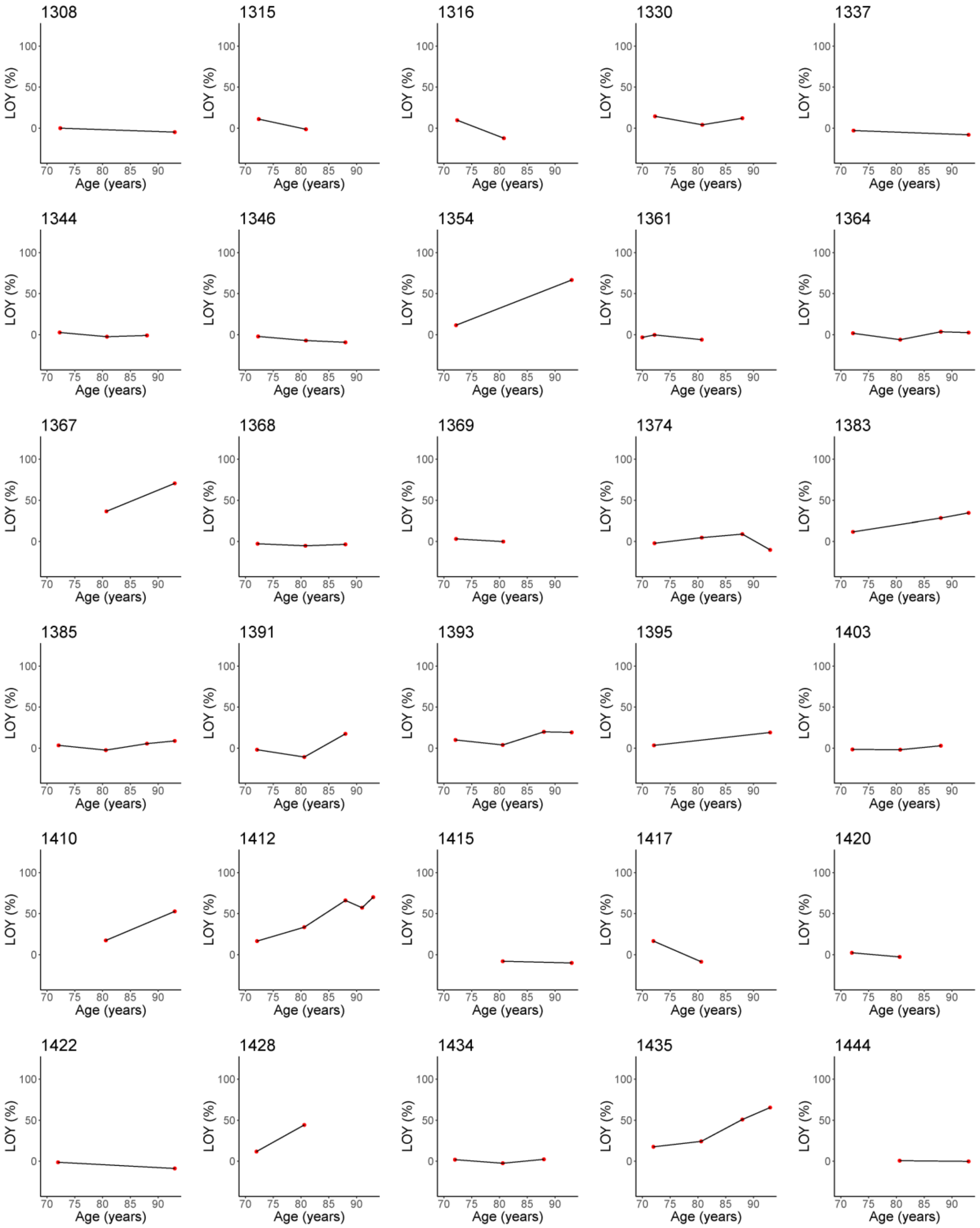

Supplementary Figure 4. (part 9/10)

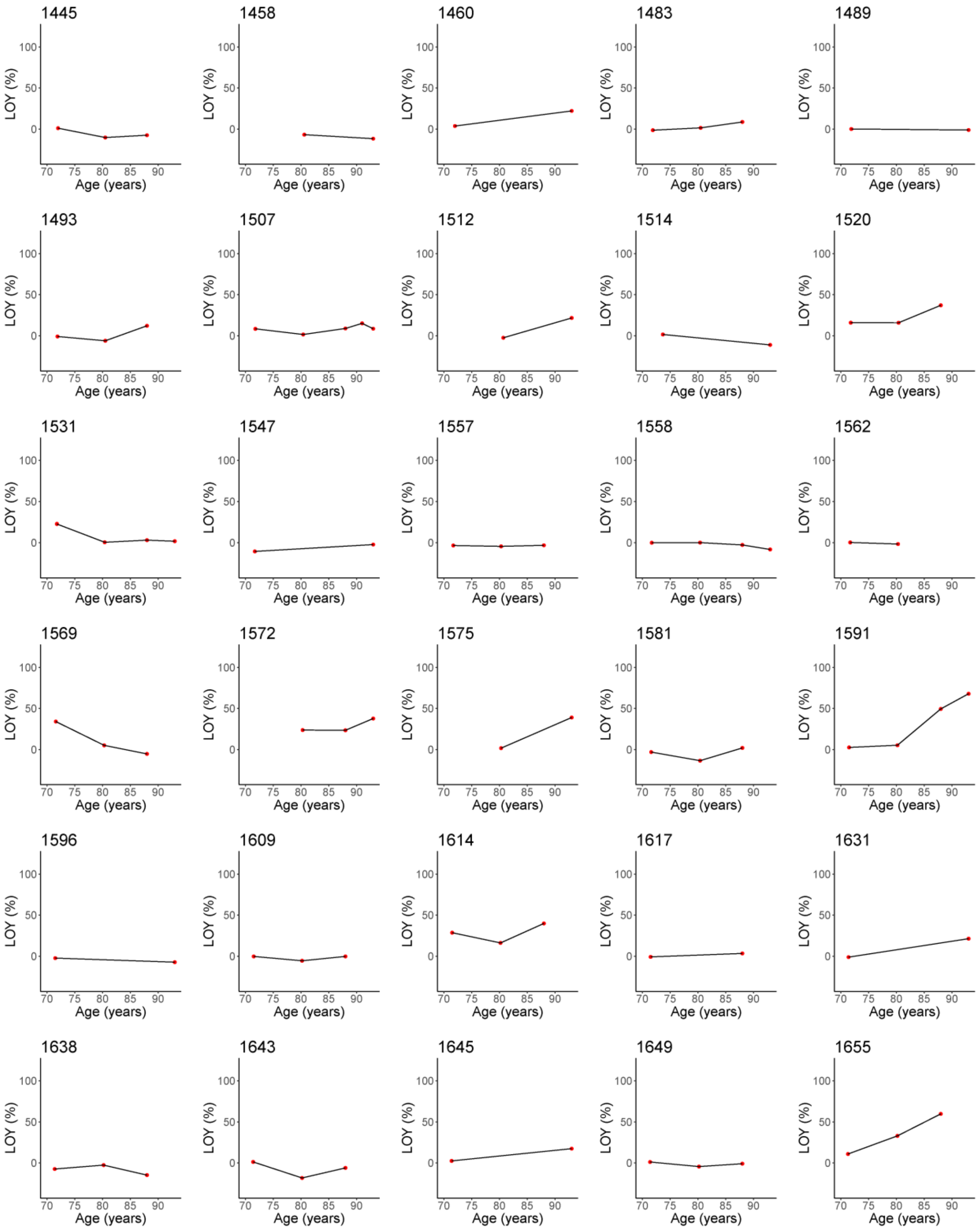

Supplementary Figure 4. (part 10/10)

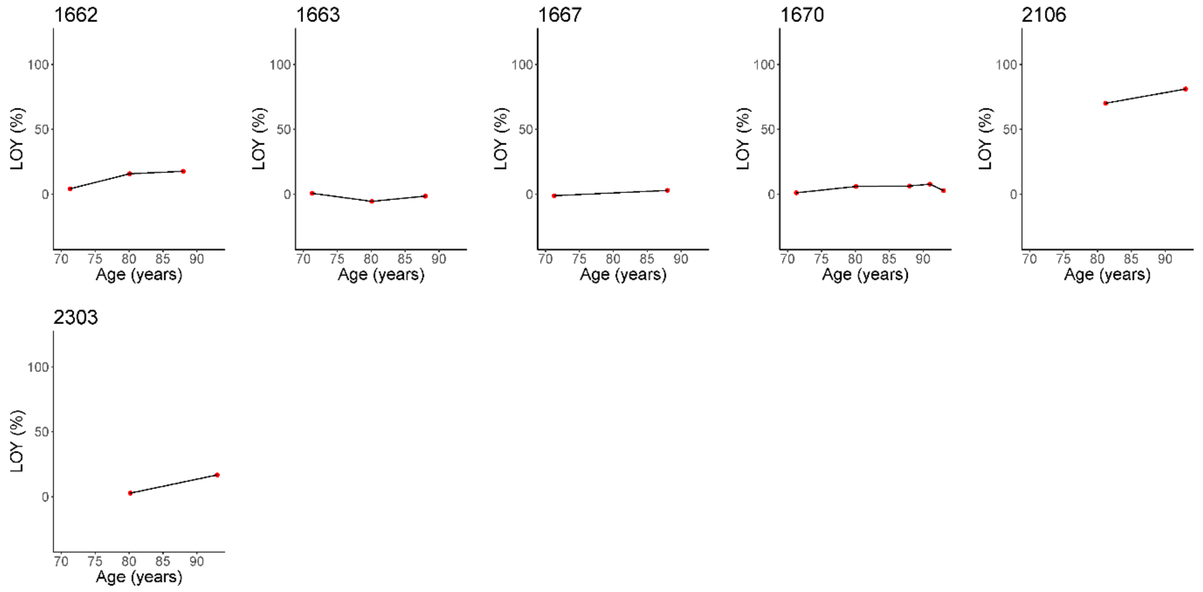

Supplementary Fig. 4. Profound variation in the longitudinal changes in frequency of LOY mosaicism in blood revealed by analyses of serially collected samples collected from 276 ULSAM subjects. Each panel represents data from one individual with the percentage of LOY and the age at blood collection plotted on the Y and X-axes, respectively. Individuals were sampled 2-5 times over a period of up to 22.2 years. The numbers above each panel represents the cohort specific individual ID-tags.

Supplementary Table 1. Examples of methods used for detection of Y aneuploidy and mosaic loss of chromosome Y (LOY).

Method	Reference
FISH	Ganster, C. et al. New data shed light on Y-loss-related pathogenesis in myelodysplastic syndromes. Genes Chromosomes Cancer 54, 717-724 (2015).
FISH	Persani, L. et al. Increased loss of the Y chromosome in peripheral blood cells in male patients with autoimmune thyroiditis. Journal of autoimmunity 38, J193-196 (2012).
FISH	Lleo, A. et al. Y chromosome loss in male patients with primary biliary cirrhosis. Journal of autoimmunity 41, 87-91 (2013).
Karyotyping	Jacobs, P.A., Brunton, M., Court Brown, W.M., Doll, R. & Goldstein, H. Change of human chromosome count distribution with age: evidence for a sex differences. Nature 197, 1080-1081 (1963).
Karyotyping	UKCCG Loss of the Y chromosome from normal and neoplastic bone marrows. United Kingdom Cancer Cytogenetics Group (UKCCG). Genes Chromosom Cancer 5, 83-88 (1992).
qPCR	Noveski, P. et al. Loss of Y Chromosome in Peripheral Blood of Colorectal and Prostate Cancer Patients. PLoS One 11, e0146264 (2016).
qPCR	Kimura, A. et al. Loss of chromosome Y in blood, but not in brain, of suicide completers. PLoS One 13, e0190667 (2018).
qPCR	Hirata, T. et al. Investigation of chromosome Y loss in men with schizophrenia. Neuropsychiatr Dis Treat 14, 2115-2122 (2018).
SNP-array & WGS	Forsberg, L.A. et al. Mosaic loss of chromosome Y in peripheral blood is associated with shorter survival and higher risk of cancer. Nat Genet 46, 624-628 (2014).
SNP-array	Loftfield, E. et al. Predictors of mosaic chromosome Y loss and associations with mortality in the UK Biobank. Sci Rep 8, 12316 (2018).
SNP-array	Grassmann, F. et al. Y chromosome mosaicism is associated with age-1 related macular degeneration. Eur J Hum Genet this issue (2018).
SNP-array & WGS	Forsberg, L.A. et al. Mosaic loss of chromosome Y (LOY) in leukocytes matters. Nature Genetics 51: 4-7 (2019).
SNP-array	Dumanski, J.P. et al. Smoking is associated with mosaic loss of chromosome Y. Science 347, 81-83 (2015).
SNP-array	Wong, J.Y.Y. et al. Outdoor air pollution and mosaic loss of chromosome Y in older men from the Cardiovascular Health Study. Environ Int 116, 239-247 (2018).
SNP-array & qPCR	Haitjema, S. et al. Loss of Y Chromosome in Blood Is Associated with Major Cardiovascular Events during Follow-up in Men after Carotid Endarterectomy. Circ Cardiovasc Genet 10:e001544 (2017).
SNP-array & WGS	Dumanski, J.P. et al. Mosaic Loss of Chromosome Y in Blood Is Associated with Alzheimer Disease. Am J Hum Genet 98, 1208-1219 (2016).
SNP-array & WGS	Wright, D.J. et al. Genetic variants associated with mosaic Y chromosome loss highlight cell cycle genes and overlap with cancer susceptibility. Nat Genet 49, 674-679 (2017).
WGS	Zink, F. et al. Clonal hematopoiesis, with and without candidate driver mutations, is common in the elderly. Blood 130, 742-752 (2017).

Supplementary Table 2. Number of probes positioned in the pseudo autosomal regions (PAR) and the male specific part of chromosome Y (MSY) on the different version of Illumina SNP-arrays used in the present study. The number in parentheses indicate the number of experiments performed using each platform in the longitudinal dataset.

SNP-array platform	Number of MSY probes	Number of PAR probes
1MDuo (n=102)	4224	832
2.5M Omni (n=341)	2494	550
Omni Express Exome (n=240)	1387	809
Infinium QC Array (n=115)	1401	545

Supplementary Table 3. Measurements of LOY in the same samples using three independent technologies, i.e. SNP-array, WGS and ddPCR (part 1/3).

ID	LOY estimated by SNP-array (mLRRY)	LOY estimated by SNP-array (%)	LOY estimated by WGS (%)	LOY estimated by ddPCR (%)
63	0,01	-1,64		4,75
80	-0,02	2,62		14,50
91	-0,08	11,03		15,40
123	-0,34	37,15	41,23	44,20
153	0,03	-3,75		5,65
207	0,03	-4,35		-13,00
241	-0,51	50,40	53,34	55,35
255	-0,03	4,16		3,40
261	-0,22	26,66	30,86	32,85
278	-0,04	5,15		16,65
300	-0,05	7,02		12,35
333	-0,78	66,26	65,61	66,20
355	-0,29	32,77	35,49	38,65
387	-0,79	66,33		66,60
419	-0,18	21,90	27,93	31,10
434	-0,06	7,91		6,25
474	-0,08	10,04	4,39	6,00
498	-0,16	20,31	26,54	28,55
504	-0,08	10,30	-0,47	1,65
506	-1,34	84,32		78,35
514	-0,48	48,60		54,75
523	-0,02	2,50		6,40
524	-0,90	71,20	67,92	71,45
529	-0,05	7,01		6,35
554	-0,03	4,71		10,35
579	-0,07	9,05	15,27	17,90
597	-0,09	11,20		21,45
622	-1,20	80,99	76,71	79,10
632	-0,02	3,11		8,35
661	-0,09	11,71		16,50
668	0,02	-2,67		7,55
674	-0,20	24,50	28,90	29,85
686	0,02	-2,22		1,20
697	-0,02	2,10		1,80
708	-0,03	4,05	3,72	5,25
729	0,01	-2,02		5,90
739	-0,04	5,43		3,40
767	-0,02	2,88		7,25
772	0,01	-1,91		6,95

Supplementary Table 3. (part 2/3)

ID	LOY estimated by SNP-array (mLRRY)	LOY estimated by SNP-array (%)	LOY estimated by WGS (%)	LOY estimated by ddPCR (%)
777	-0,13	17,05		21,85
810	-0,29	32,69		37,90
812	-0,14	17,43		19,10
826	-0,41	43,46	45,97	47,00
838	0,00	-0,60		6,15
850	-0,30	34,03		39,80
884	-0,06	7,36		18,90
896	-0,19	22,97		32,05
897	-0,01	0,85		3,65
908	0,00	-0,50		4,50
920	0,00	0,57		7,50
955	0,02	-3,39		-0,50
973	-0,37	40,18	42,51	44,80
980	-0,20	24,05		22,60
984	-2,00	93,77		90,35
997	-0,04	5,79		4,00
1006	0,04	-5,51		3,00
1019	-0,11	14,26		7,10
1022	-0,01	1,38		10,50
1026	-0,11	13,74		27,05
1032	-0,09	11,48		21,20
1034	0,02	-3,01		3,20
1035	0,03	-3,86		5,80
1060	-0,07	8,96		18,85
1062	0,01	-1,60		8,55
1074	-0,97	74,09	67,35	67,35
1076	-0,10	12,47		14,70
1085	-0,02	2,76		9,30
1099	0,04	-5,19		3,15
1103	-0,19	23,46		36,05
1124	-0,07	9,61	19,37	21,30
1127	-0,03	4,50		5,00
1149	0,00	-0,68		1,85
1162	-0,61	57,30	59,84	64,00
1181	0,04	-6,14		0,00
1191	-0,05	6,63		0,00
1202	-0,04	5,89		2,00
1208	-0,02	3,26	8,64	13,15
1222	-0,54	52,83		60,00

Supplementary Table 3. (part 3/3)

ID	LOY estimated by SNP-array (mLRRY)	LOY estimated by SNP-array (%)	LOY estimated by WGS (%)	LOY estimated by ddPCR (%)
1223	-0,05	7,05		17,30
1226	0,03	-4,20		3,90
1252	-0,05	6,82		18,45
1270	-0,17	20,60		32,50
1272	-0,35	38,53	42,73	43,35
1276	-0,73	63,82		65,90
1295	0,02	-3,27		4,20
1308	-0,02	2,23		7,10
1337	0,01	-0,81		3,60
1354	-0,84	68,92		69,80
1364	-0,07	9,13		22,80
1367	-0,93	72,58		73,35
1374	0,02	-2,71		7,55
1383	-0,36	39,04	44,35	47,10
1385	-0,12	14,93		22,55
1393	-0,20	24,58		31,50
1395	-0,20	24,52		37,80
1410	-0,59	55,95		57,40
1412	-0,92	72,15	72,40	74,25
1415	0,02	-2,63		2,10
1422	0,01	-1,43		1,15
1435	-0,82	67,82	67,58	70,20
1444	-0,05	6,62		15,45
1458	0,03	-4,18		-0,25
1460	-0,23	27,34		38,15
1489	-0,04	5,66		11,40
1507	-0,12	14,78		22,70
1512	-0,23	26,98		38,20
1514	0,03	-3,58		6,50
1531	-0,06	8,50		8,35
1547	-0,03	4,72		9,35
1558	0,01	-0,84		2,95
1572	-0,39	41,95	45,21	46,35
1575	-0,41	43,10		50,30
1591	-0,87	70,08	68,30	69,35
1596	0,00	0,03		6,75
1628	-0,01	0,78		6,20
1631	-0,22	26,68		36,50
1645	-0,19	23,03		34,35
1670	-0,07	9,32		17,70
1834	-0,02	3,13		4,05
2106	-1,25	82,31		80,75
2303	-0,18	22,34		36,05
